# MAINTAINING BRAIN HEALTH VIA TELEHEALTH-BASED CHAIR YOGA FOR RURAL UNDERSERVED OLDER ADULTS

**DOI:** 10.1093/geroni/igad104.0520

**Published:** 2023-12-21

**Authors:** JuYoung Park, Lisa Ann Wiese, Janet Holt

**Affiliations:** Florida Atlantic University, Boca Raton, Florida, United States; Florida Atlantic University, Boca Raton, Florida, United States; Florida Atlantic University, Boca Raton, Florida, United States

## Abstract

We conducted a randomized control pilot trial in an underserved, racially/ethnically diverse community, to examine the efficacy of (1) computer literacy training provided by high school students meeting rural older adults in their homes, followed by 2) telehealth-based online chair yoga (OCY) or computer brain games (CBG). First, high school students engaged in a “train the trainer” computer skills course using a previously-tested curriculum designed for the target population. Students mentored the older adults in learning computer skills and to access either the OCY (n = 15) or CBG (n=15), which were randomly assigned. Outcomes measured at pre, post, and three months following the 12-week intervention included computer proficiency, cognitive function, pain levels, and psychosocial well-being. In a linear mixed growth model with random intercepts, there was a significant linear trend in computer-based competency, t(50, 19) = 2.56, p = .013. Computer proficiency (computer basics, Internet and email use, communication, and calendaring) increased significantly in both the OCY and CBG groups. Importantly, there was a significant linear change in pain by group, controlling for age and living alone, F(1, 14) = 6.64, p = .022, η2 = .32 (large effect size). Chronic pain in the OCY group decreased significantly from baseline to 3-month follow-up (18.89 to 11.35) but increased in the CBG group (14.27 to 17.28). Increases in cognitive function increased pre/post t(29) = -2.98, p = .003 (one-tailed). These results are promising for older adults with limited exercise opportunities, who also face cognitive risk exacerbated by elevated pain levels.

